# Endonuclease V activated *Pyrococcus furiosus* Argonaute for the detection of food contaminated bacteria

**DOI:** 10.1038/s41538-025-00675-6

**Published:** 2025-12-31

**Authors:** Yiheng Shi, Pei Gao, Di Wu, Yongning Wu, Guoliang Li

**Affiliations:** 1 School of Food Science and Engineering, Shaanxi University of Science & Technology, Xi’an, PR China; 2 Institute for Global Food Security, School of Biological Sciences, Queen’s University Belfast, Belfast, UK; 3 NHC Key Laboratory of Food Safety Risk Assessment, Food Safety Research Unit (2019RU014) of Chinese Academy of Medical Science, China National Center for Food Safety Risk Assessment, Beijing, China

**Keywords:** Biological techniques, Biotechnology, Cancer, Microbiology, Molecular biology

## Abstract

*Pyrococcus furiosus* Argonaute (*Pf*Ago) is a novel programmable nuclease that has been used in nucleic acid detection due to its excellent performance. Traditional *Pf*Ago based detection methods relies on the input of exogenous guide DNA (gDNA), which restricted its flexibility and universality in application. Here, we designed primers with deoxy inosine base which can be recognized and cleaved by endonuclease V, following turn into gDNA to activate *Pf*Ago for target gene detection. Therefore, an Endo V activated *Pf*Ago based nucleic acid detection (VPN) method was developed. The detection limit of this method was 0.04 ng/μL DNA. Moreover, the method was successfully applied to the detection of food contaminated bacteria. This approach provided a universal and power tool for the detection of nucleic acid-containing organisms such as pathogens, viruses, and tumor cells.

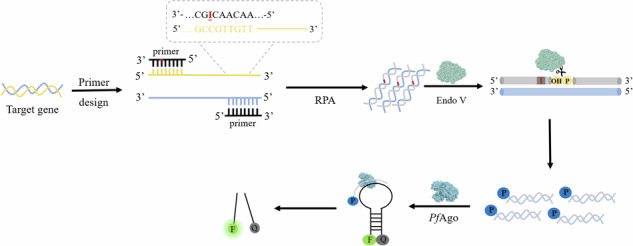

## Introduction

The frequent occurrence of food safety problems caused by pathogenic microorganisms in food poses a serious threat to human health and the development of food industry. Pathogenic bacteria such as *Salmonella typhimurium*, *Escherichia coli* O157: H7, *Staphylococcus aureus* and *Cronobacter sakazakii* are common foodborne pathogens that cause outbreaks of foodborne diseases, resulting in serious mortality and economic losses^1–3^. Food-borne pathogens are prone to contaminate a variety of foods, such as animal foods represented by meat, eggs, milk, and their products^4^. Environmental pollution in food production, processing, transportation, sales, and other links may also lead to foodborne pathogen contamination^5^. Ingestion of food contaminated by foodborne pathogens can lead to serious health problems, such as recurrent intestinal inflammation, diarrhea, vomiting, chronic kidney disease, and even death^6,7^.In addition to pathogenic bacteria that endanger human health, some spoilage microorganisms have a negative impact on the quality of food such as odor, color, and taste by producing odor, gas, and mucus, thereby affecting the shelf life and sales market^8^. Some microorganisms also produce some harmful substances in the metabolic process, accumulate in food, and then endanger human health, such as bacterial toxins, biogenic amines, etc^9,10^. Therefore, the timely and accurate detection of contaminated bacteria in food has become an urgent problem to be solved in the field of food safety.

Nucleic acid detection, such as Polymerase chain reaction (PCR) is a very classic and widely used technique in molecular biology. The principle is to use specific primers to exponentially amplify the unique and conserved DNA fragments of the target bacteria in vitro, and then detect the PCR products by agarose gel electrophoresis or real-time fluorescence to determine whether the food contains the bacteria. However, PCR also has the disadvantages of time-consuming, high equipment cost, and inability to quantitatively detect. There are also emerging nucleic acid detection methods in recent years, the use of programmable nucleases to detect nucleic acids of targets, such as clustered regularly interspaced short palindromic repeats (CRISPR)-related proteins (Cas)^11^,and *Pyrococcus furiosus* Argonaute (*Pf*Ago) endonuclease derived from *Pyrococcus furiosus*^12^. *Pf*Ago is used for DNA detection and gene editing. It can cleave the target single-stranded DNA with a small fragment of 5’ -phosphate terminal ssDNA as gDNA, and cut the phosphate diester bond between the 10th and 11th nucleotides of the target DNA from the 5’ end. The resulting single-stranded DNA can be used as a second gDNA to guide *Pf*Ago for secondary cleavage^13,14^. Compared with Cas enzyme, the activity of *Pf*Ago does not depend on the PAM sequence, and its built-in signal amplification function makes the *Pf*Ago-based biosensor more flexible in nucleic acid detection applications. At present, several detection methods based on *Pf*Ago have been developed to detect organisms such as Goose parvovirus (GPV), *Listeria monocytogenes*, *Salmonella*, etc^15–17^. However, *Pf*Ago cannot exert its maximum cutting ability due to its dependence on the input of exogenous gDNA.

Here, Endo V activated *Pf*Ago based nucleic acid detection (VPN) method was developed for food contaminated bacteria analysis. In this system, target gene was amplified with forward primer containing a deoxy-inosine (dI) base. Through the recognition and shearing effect of the Endo V enzyme, a phosphate group was generated at the 5’ end, following turns into ssDNA at high temperature, acting as gDNA to activate *Pf*Ago. Finally, a molecule beacon (MB) with fluorophore and quencher at the two ends was cleaved, resulting in fluorescence recovery. The results can be monitor by fluorescence spectrophotometer or visual observation using a portable ultraviolet lamp. This approach gets rid of the requirement of exogeneous gDNA in traditional *Pf*Ago based nucleic acid detection method, showing excellent flexibility and applicability. The detection system can not only meet need of common food contaminated bacteria detection, but also provide a new idea for the sensitive and efficient detection of nucleic acid-containing organisms such as fungus, viruses and tumor cells.

## Results and discussion

### Principle of VPN

**Fig. 1:**
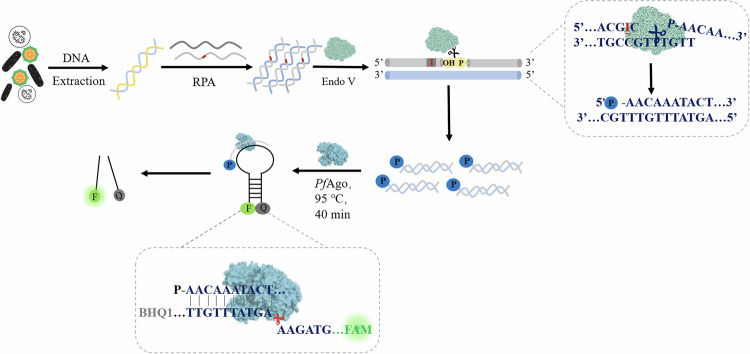
Schematic illustration of Endo V activated *Pf*Ago detection method. (source: Microsoft Office PowerPoint).

In the previously reported *Pf*Ago-based detection system, a short ssDNA with phosphate group at 5’ end was need to act as gDNA^18^. The phosphorylated process was generally conducted separately before the detection start. Therefore, the cleavage activity of *Pf*Ago is highly depend on the phosphorylation efficiency of ssDNA. In order to overcome this drawback, we proposed an endogenous gDNA generation strategy. As shown in Fig. 1, the normal base (dT or dG) at specific site of one primer was substituted by the deoxy inosine (dI) base. After amplification, the target double strand gene containing dI base can be recognized by Endo V. Then, the Endo V cleaved the phosphate diester bond of the second base adjacent to deoxy inosine nucleosides, resulting in a gap of 3’-hydroxyl and 5’-phosphate. The fragment with a 5’-phosphate terminal can convert to ssDNA at high temperatures, acting as gDNA and activating the *Pf*Ago-mediated cleavage reaction. Finally, the MB ring complementary to gDNA was cleaved, resulting in fluorescence recovery. Therefore, with the help of Endo V, endogenous gDNA is generated to activate *Pf*Ago for target gene detection. In this strategy, there are no special requirement for the target gene sequence (such as PAM site or restriction endonuclease site) or complex primer and probe design. showing more versatile and flexible in nucleic acid detection.

### Feasibility of VPN

**Fig. 2:**
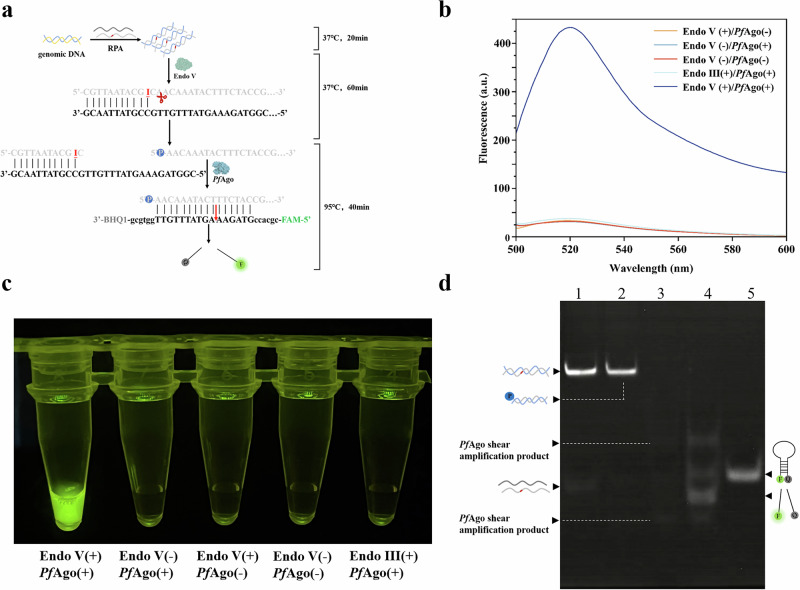
Feasibility verification. **a** Sequence cut diagram of VPN. P: phosphorylation. F: FAM fluorophore. Q: BHQ1 quencher. **b** Fluorescence spectra of different enzyme systems. **c** Fluorescence imaging of different systems at 365 nm ultraviolet light. ‘+’ represents the presence of *Pf*Ago or Endo V or Endo III, and ‘-’ represents the absence of *Pf*Ago or Endo V or Endo III. **d** Native polyacrylamide gel electrophoresis (15%). (line 1:amplification product, line 2: Endo V cleave amplification product, line 3: in the absence of MB, *Pf*Ago cleave Endo V cleavage products, line 4: *Pf*Ago cleave Endo V cleavage products with MB, line 5: MB). (source: Microsoft Office PowerPoint, Origin).

In order to evaluate the feasibility of the detection system, *E. coli* O157:H7 was selected as the conceptual verification target. Firstly, the genomic DNA was extracted as the template. After amplification, the fragment containing dI base could be recognized and cleaved by Endo V, therefore, activating *Pf*Ago to cleave MB (Fig. 2a). As shown in Fig. 2b, when endonuclease III (Endo III) was added to the system instead of Endo V, there was no change in fluorescence intensity. In the absence of Endo V or *Pf*Ago, the fluorescence intensity of the system also did not change significantly. When Endo V and *Pf*Ago coexist in the detection system, a significant change in fluorescence intensity could be observed. The same phenomenon can also be seen in Fig. 2c, strong fluorescence appears only when Endo V and *Pf*Ago coexist in the system. While in other cases, fluorescence cannot be observed by the naked eyes under 365 nm ultraviolet light. The reason why Endo III cannot replace Endo V to make the system emit fluorescence is that the specific recognition site of Endo III is different from that of Endo V. Endo V can recognize the dI base in DNA, that is, the deamination product of deoxyadenosine. Endo III has both N-glycosidase activity and AP-lyase activity, but the bases it can recognize and cut do not include the dI base^19,20^. At the same time, we also introduced a control group without dI bases in the amplification primers, and performed VPN detection on *E. coli* O157:H7 (10^3^, 10^4^, 10^5^,10^6^ CFU/mL) at different concentrations (Fig. S1). The VPN system without dI bases in the amplification primers is almost non-fluorescent. The above results show that the existing detection methods are feasible and have good responsiveness.

Next, we verified the feasibility of the VPN detection system by native-PAGE (Fig. 2d, Supplementary Fig. S2). The band on Lane 1 represents the RPA amplification product, and the band on Lane 2 represents the RPA amplification product cleaved by Endo V. The Endo V cut amplification product is actually not much different from the RPA amplification product, so the band mobility of Line 1 and Line 2 is similar. Lane 3 corresponds to *Pf*Ago cutting the Endo V cut product without the presence of MB. Lane 4 corresponds to the cleavage product of Endo V by *Pf*Ago in the presence of MB, and the band on Lane 5 represents a separate MB. The bands with similar migration rates in Line 3 and Line 4 should be obtained by *Pf*Ago cutting RPA amplification products. This part of *Pf*Ago cutting accounts for a relatively small proportion, so the band color is lighter. The bands with similar migration rate in Line 4 and Line 5 are complete MB, and the bands with faster migration rate than MB in Line 4 are MB products cut by *Pf*Ago. These gel results also confirmed again that the VPN detection method proposed in this study is feasible.

### Optimization of VPN

**Fig. 3:**
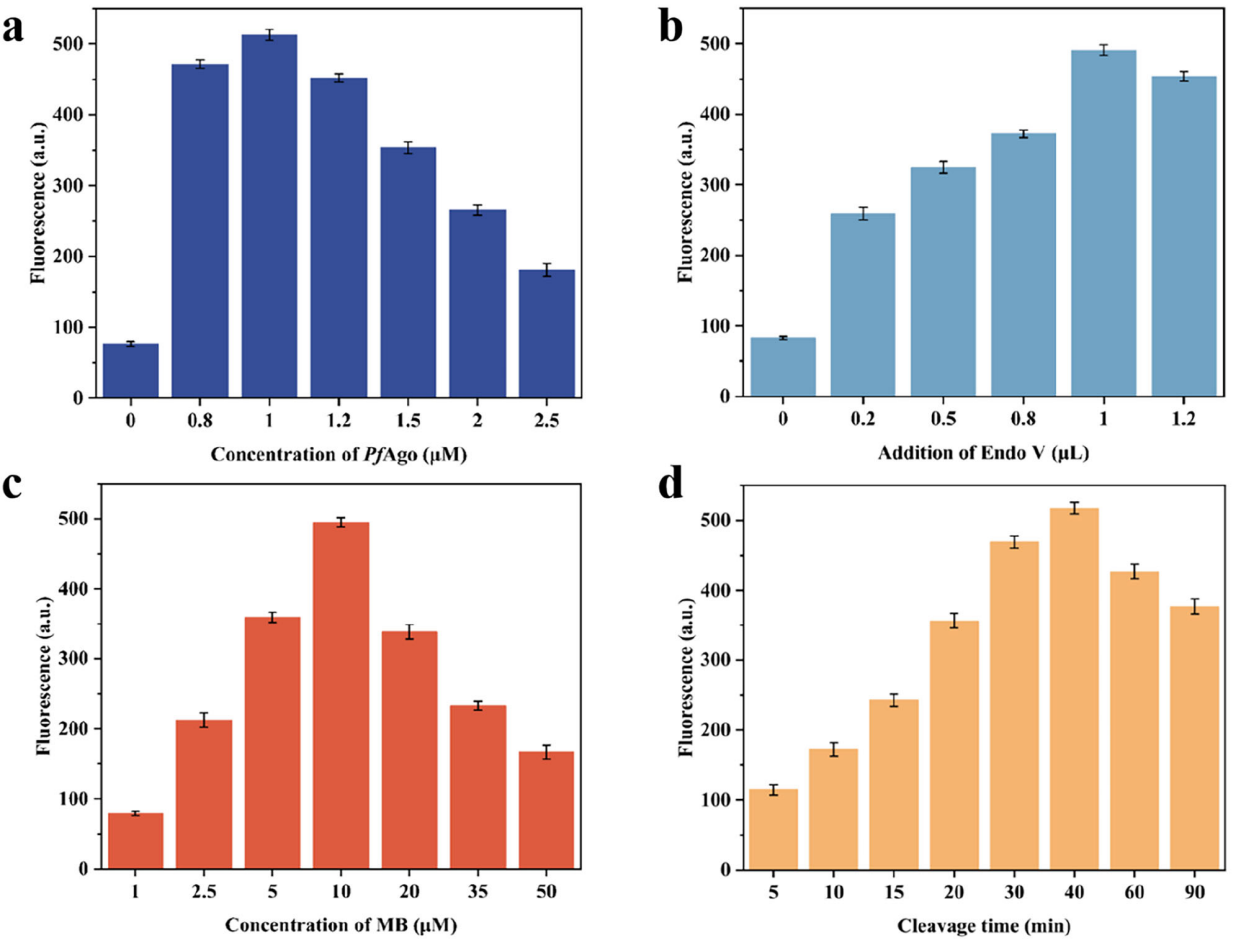
Experimental parameters optimization of VPN. **a** Optimization of *Pf*Ago concentration. **b** Optimization of the addition of Endo V. **c** Optimization of MB concentration. **d** Optimization of cleavage time. (source: Microsoft Office PowerPoint, Origin).

Next, in order to make the detection system achieve the best detection performance, we optimized the parameters that affect the detection performance of VPN. As shown in Fig. 3a, compared with the blank group, when 0.8 μM *Pf*Ago was added to the system, the fluorescence intensity was significantly enhanced. When the concentration of *Pf*Ago increased to 1 μM, the fluorescence intensity of the system reached the maximum. However, when the concentration of *Pf*Ago continued to increase, the corresponding fluorescence intensity showed a slow downward trend. These phenomena may be due to the following reasons: (i) at 1 μM, the enzyme was saturated with all available target nucleic acids, and the signal amplification efficiency was maximized. Excessive *Pf*Ago may lead to space competition between enzyme molecules and hinder the formation of effective complexes. (ii) above this concentration, excessive *Pf*Ago will induce non-specific cleavage and increase the negative control signal. (iii) As a protein, although *Pf*Ago has not been specifically reported, it may still undergo specific aggregation at high concentrations, thereby reducing its cleavage efficiency. Therefore, 1 μM was selected as the optimal reaction concentration of *Pf*Ago in this experiment.

Secondly, the addition amount of Endo V (enzyme activity) was optimized so that the system could produce more endogenous gDNA. It can be seen from Fig. 3b that compared with the blank group, the fluorescence intensity of the system gradually increased with the addition of Endo V. When the addition amount of Endo V was 1.0 μL, that is, the enzyme activity was 10 U/μL, the fluorescence intensity was the largest, and then gradually decreased. Therefore, the optimal addition amount of Endo V in the detection system was 1.0 μL (the enzyme activity was 10 U).

The reaction concentration of MB also affects the detection performance of this method. As shown in Fig. 3c, with the increase of MB concentration in the detection system, the corresponding fluorescence intensity showed a trend of increasing first and then decreasing. When the concentration of MB was greater than 10 μM, the corresponding fluorescence intensity showed a significant downward trend. When the concentration of MB was 20 μM and 35 μM, the fluorescence intensity decreased by nearly twice compared with that when the concentration of MB was 10 μM. Therefore, we chose 10 μM MB concentration for further study.

Finally, in order to determine the optimal reaction time of the detection system, we set several groups of reaction time as shown in Fig. 3d to test the change of fluorescence intensity with *Pf*Ago cutting time to improve the detection performance of the detection system. From the diagram, it can be observed that the fluorescence intensity first showed a rapid growth trend with time, and the growth rate of the reaction rate slowed down after 30 min, and it was still rising slowly within 30–40 min, and then showed a downward trend. Therefore, the optimal reaction time of the detection system was determined to be 40 min. Under the above optimal conditions, the established the detection system can obtain the best performance.

### Performance of VPN

**Fig. 4:**
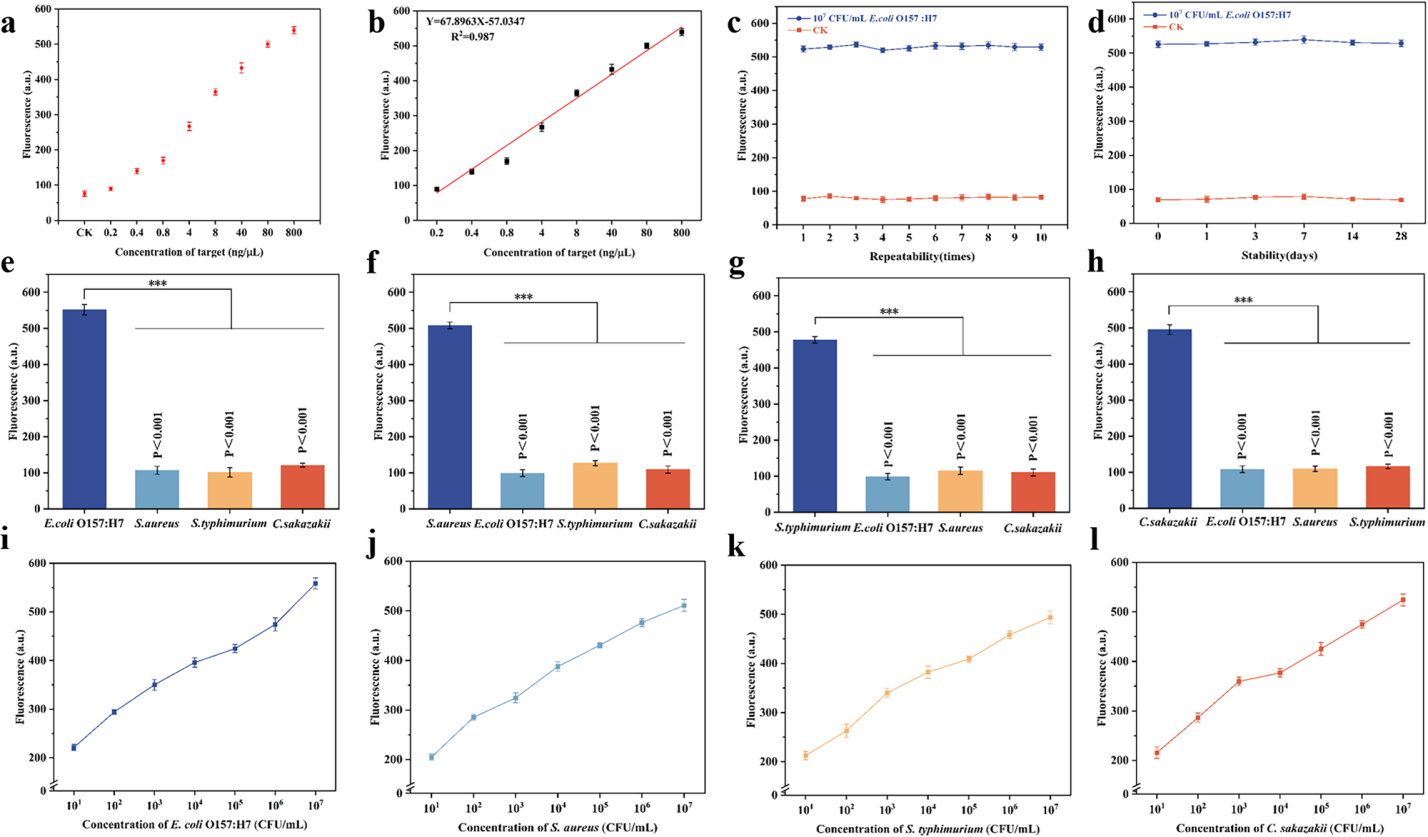
Use VPN to detect a single foodborne pathogen. **a** Detection of DNA by VPN. **b** A linear regression equation with a target gene concentration of 0.2-800 ng/μL. **c** The repeatability of VPN. **d** Stability of VPN. VPN for the detection of the four food-borne pathogens : *E. coli* O157:H7 (**e**), *S. aureus* (**f**), *S. typhimurium* (**g**) and *C. sakazakii* (**h**),under different types of interference. The concentration of non-target bacteria is 100-fold higher than that of target bacteria. Date are presented as mean ± s.d., *n* = 3. *P* value were calculated using two-sided one-way ANOVA post-Dunnett’s test;***p < 0.001. **i–l** The sensitivity of VPN to detect four food-borne pathogens : *E.coli* O157:H7 (**i**), *S.aureus* (**j**), *S. typhimurium* (**k**) and *C. sakazakii (***l**). (source: Microsoft Office PowerPoint, Origin).

In order to illustrate the performance of the detection method, we used Dnase/Rnase-free deionized water to gradient dilute genomic DNA of the target strain (*E. coli* O157:H7) and performed RPA amplification to obtain 0–800 ng/μL of the sample to be tested. The optimal system was used to detect nucleic acid products at different concentrations. The detection results were shown in Fig. 4a, and the fluorescence intensity of the system increases with the increase of the target concentration. A linear relationship was established for the fluorescence intensity at 520 nm again the concentration of amplification products (Fig. 4b). The concentration of the target DNA showed a good linear relationship with the fluorescence intensity at 0.2-800 ng/μL. Therefore, the linear regression equation of the target DNA was Y = 67.8963 * X - 57.0347 (R^2^ = 0.987), where X and Y represent the concentration and fluorescence intensity of the target DNA respectively. The detection limit of this method is 0.04 ng/μL DNA, calculated at the concentration corresponding to the fluorescence intensity of 3 standard deviation of the control group without DNA. This indicates that the detection method of Endo V activated *Pf*Ago we constructed has good responsiveness to the target bacteria. In addition, we evaluated the repeatability of the method (Fig. 4c). Regardless of whether the operator is replaced or not, the method can consistently distinguish negative and positive samples. These results highlight the excellent repeatability of the detection system developed in this study. Finally, we evaluated the stability of the detection method, we carried out the real-time stability verification experiments based on time gradient. It can be seen that the detection system still maintains excellent performance after 28 days. (Fig. 4d).

Afterwards, we evaluated the specificity of the detection system by using the VPN detection system to detect a variety of bacteria, including *E. coli* O157:H7, *S. typhimurium*, *S. aureus* and *C. sakazakii*, as shown in Fig. 4e–h. This method uses specific amplification primers and four corresponding MBs. It can be observed from the figure that only fluorescence appears in the amplification system using primers corresponding to the target bacteria. The above results show that the detection method established in this study can accurately identify the target strain.

Under the optimized parameters, we tested the sensitivity of the VPN method to four foodborne pathogens: *E. coli* O157:H7, *S. typhimurium*, *S. aureus* and *C. sakazakii*. As shown in Fig. 4i–l, the fluorescence intensity increased with the increase of the concentration of foodborne pathogens, indicating that the VPN method successfully detected the lower concentration range of all four foodborne pathogens from 10^1^ CFU/mL to 10^7^ CFU/mL. Therefore, the LOD of the detection system was 10^1^ CFU/mL. The results showed that we successfully constructed an Endo V activated *Pf*Ago detection method for food-borne pathogens.

Yu^21^ and others established the reverse transcription loop-mediated isothermal amplification (RT-LAMP) combined with *Pf*Ago for detecting Porcine epidemic diarrhea virus (PEDV). This method requires the design of five amplification primers and three gDNA, and gDNA needs to be phosphorylated by T4 polynucleotide kinase (T4 PNK). Pang et al.^22^ developed the handheld isothermal nucleic acid amplification device (WeD-1) combined with LAMP-*Pf*Ago nucleic acid detection method. This detection method requires the design of 6 amplification primers and 2 phosphorylated gDNA. As well as the previously proposed *Pf*Ago-based detection method (PAD) which requires the use of T4 PNK to phosphorylate gDNA in advance^23^. This process takes at least 30–60 min which make the detection time longer. The primer design of the method proposed in this study is simpler and more convenient. It is only necessary to design a set of amplification primers with dI bases, and then endogenously generate gDNA through the recognition and cleavage of Endo V, which can activate the cleavage activity of *Pf*Ago. Therefore, the detection method proposed in this study has certain advantages compared with some detection strategies currently used to detect food contaminated bacteria (Table 1). At the same time, thanks to the signal amplification function of RPA and *Pf*Ago, our method shows good analytical performance in ultrasensitive detection, and the ingenious primer design makes the method have the potential to be applied to more targets.

**Table 1:** Comparison of different nucleic acid detection method for food contaminated bacteria

description	LOD (CFU/mL)	specificity	detection time	gDNA	References
Magnetic separation and HCR^a^	1.68	yes		no	^25^
Colorimetric DNAzyme Biosensor	100	yes		no	^26^
PAD^b^	10^1^	yes		external input	^23^
EGG-PAD^c^	5.7 × 10^1^	yes	2–3 h	endogenous generate	^24^
LAMP with *Pf*Ago^d^	10^0^	yes	50 min	yes	^27^
PCR-*Pf*Ago	2.7 × 10^0^	yes		yes	^12^
IMS-PMAxx-ddPCR^e^	5.6 copies/g	yes		no	^28^
RT-PSR and Hydroxynaphthol Blue Indicator	1.2 × 10^1^	yes	55 min	no	^29^
VPN^f^	10^1^	yes	2 h	**endogenous generate**	this work

^a^Hybridization chain reaction.

^b^*Pf*Ago based *A. acidoterrestris* detection.

^c^Enzyme-assisted endogenous gDNA generation to activate *Pf*Ago for *A. acidoterrestris* detection.

^d^loop-mediated isothermal amplification with *Pyrococcus furiosus* Argonaute.

^e^Immunomagnetic separation-propidium monoazide-droplet digital PCR.

^f^Endo V activated *Pf*Ago based nucleic acid detection.

### Multiple detection applications and real sample analysis

**Fig. 5:**
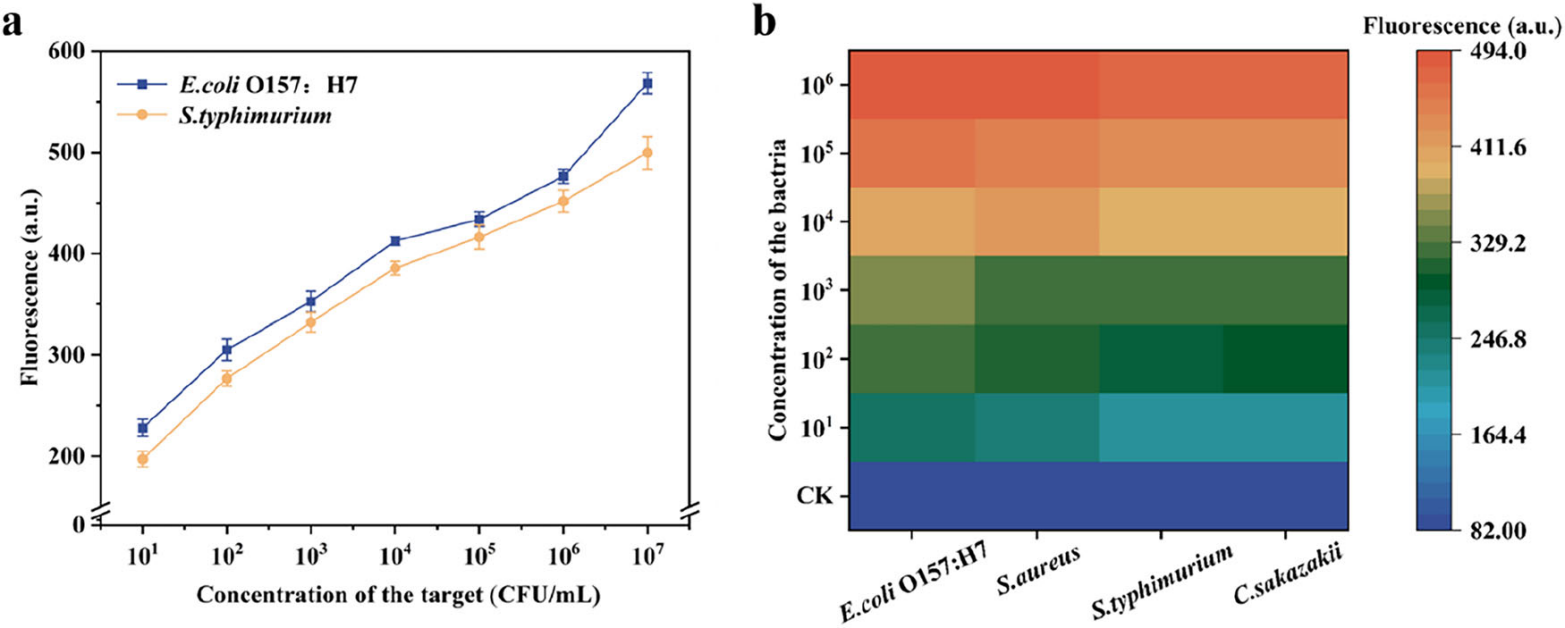
Performance of VPN. **a** VPN for multiplex detection of two foodborne bacteria. **b** The detection performance of VPN in real samples (source: Microsoft Office PowerPoint, Origin).

We further explored the application of VPN in the multiple detection of food-contaminated bacteria. We designed MB with two different modifications, and used VPN to simultaneously detect *E. coli* O157:H7 and *S. typhimurium*. From Fig. 5a, it can be seen that VPN can detect two targets in a single reaction, and the intensity of the two output signals in the reaction is at a similar level. Therefore, VPN has the ability to simultaneously detect two or more food-contaminated bacteria.

Next, in order to evaluate the applicability of VPN in detecting foodborne pathogens in spiked real samples (beef, milk), we used different concentrations of foodborne pathogens (10^1^–10^6^ CFU/mL) to measure the fluorescence spectra of the VPN reaction system in beef and milk samples (Fig. 5b). Uninoculated samples were used as blank controls. It can be observed that the fluorescence intensity of contaminated samples (orange to red) was significantly higher than that of the control group (blue). The visualized fluorescence image (Fig. S3a) can also observe good fluorescence reaction, which is consistent with the detection results of qPCR (Fig. S3b–e). The detection results show that the VPN method has good analytical performance and can accurately and quickly detect foodborne pathogens in actual food samples.

In this study, we proposed a fluorescent detection method for detecting food contaminated bacteria in food by Endo V-activated *Pf*Ago, named VPN. This method can detect two or more food contaminated bacteria at the same time. Through ingenious design, the dI base is inserted into the amplification primer to generate gDNA endogenously, and *Pf*Ago is activated for nucleic acid detection, so that *Pf*Ago overcomes the limitation of exogenous gDNA input, thereby achieving its maximum cutting ability and detection performance. The method can detect 10^1^ CFU/mL *E. coli* O157:H7 and 0.563 ng/μL DNA. It is shown that VPN has good specificity, sensitivity and versatility, and it also shows superior performance in actual sample analysis. Therefore, this method not only expands the generation pathway of gDNA, but also provides ideas for solving the limitations of *Pf*Ago in nucleic acid detection, and shows that the method has a good application prospect in the development of nucleic acid target detection system and sensitivity detection.

## Methods

### Bacterial strains and chemicals

All strains used in the laboratory were purchased from American Type Culture Collection (ATCC, USA). All oligonucleotides and MB were designed by Premier 5.0 software (Table S1) and synthesized by Sangon Biotech Co., Ltd. (China). Restriction endonuclease was purchased from New England Biolabs (Beverly, MA,USA). The E.Z.N.A bacterial DNA kit was purchased from Omega Bio-tek, Inc. (USA). TwisDx TwistAmp®Basic kit was purchased from Shanghai Nonin Biological Technology Co., Ltd (China).

### Expression and purification of *Pf*Ago

The *Pf*Ago used in this study comes from laboratory expression and purification^24^.

### Operational workflow of VPN

Firstly, the bacterial genomic DNA was extracted, and the dI base was inserted into the amplification primer by design (F:5’-CGTTAATACGICAACAAATACTTTCTACCG-3’) and the recombinant enzyme polymerase amplification (RPA) was performed. The obtained amplification product (6 μL) with dI base was evenly mixed with 1 μL Endo V (10 U/μL) and 1 μL 10×Endo V buffer, supplemented with non-enzymatic water to 10 μL, and incubated at 37 °C for 60 min. After the completion of the reaction, 1 μM *Pf*Ago protein and 10 μM MB were added to the above system, supplemented with non-enzymatic water to 20 μL, and incubated at 95 °C for 40 min. The fluorescence intensity of the mixed system was recorded at room temperature.

### Sensitivity and specificity of VPN

To determine the sensitivity of DNA detection of VPN, bacterial genomic DNA was diluted 10-fold and amplified by RPA, following conducted VPN procedure. To the determine the sensitivity of bacteria detection, the genomic DNA of different concentrations of bacteria was extracted and amplified by RPA, following conducted VPN procedure. In order to prove the specificity of the detection method, genomic DNA of different bacteria were extracted and amplified using one same primer pair and detected by VPN.

For multiple detection of food contaminated bacteria: Firstly, bacterial genomic DNA was extracted, and 10 μL of the mixed amplification product obtained by RPA after RPA was mixed with 2 μL Endo V and 2 μL 10 × Endo V buffer, and incubated at 37 °C for 60 min. Then 1 μM *Pf*Ago and 10 μM MB (FAM-modified MB, λex: 495 nm, λem: 520 nm; ROX- modified MB, λex: 575 nm, λem:  602 nm) was incubated at 95 °C for 40 min, and the fluorescence intensity of the mixed system was recorded.

### Real sample analysis

In order to evaluate the applicability of our method in real-world scenarios, we purchased beef and milk from a local supermarket. *E. coli* O157: H7, *S. typhimurium*, *S. aureus* and *C. sakazakii* were cultured overnight under suitable conditions (FAM-modified MB, λex: 495 nm, λem: 520 nm; JOE modified MB λex: 529 nm, λem: 550 nm). Beef samples were grinded into fine powder by liquid nitrogen grinding method. The obtained samples were mixed with PBS vortex, filtered and centrifuged (12,000 rpm, 5 min, 4 °C). The supernatant was used as the processed meat sample. The *E. coli* O157: H7 and *S. typhimurium* obtained by overnight culture were gradient diluted with the supernatant, and then the DNA was extracted with the reagent box. In addition, the milk sample was diluted 10 times and centrifuged, and the supernatant was taken as the treated milk sample. The obtained supernatant was gradient diluted with *S. aureus* and *C. sakazakii* to obtain emulsion samples containing different concentrations of pathogenic bacteria, and then DNA was extracted with a reagent box. Then the VPN method was conducted to detect the samples.

## Supplementary information

Supplementary information

## Data Availability

All datasets generated or analyzed during this study are available from the corresponding author upon reasonable request.
