# Supplementary material for: Endonuclease V activated Pyrococcus furiosus Argonaute for the detection of food contaminated bacteria
Source: NPJ Sci Food. 2025 Dec 31;10:27. doi: 10.1038/s41538-025-00675-6 (PMC12859123; doi:10.1038/s41538-025-00675-6)
Supplement: Supplementary file 1 — Supplementary information [file 41538_2025_675_MOESM1_ESM.docx]

**Supporting Information**

Endonuclease V actived *Pyrococcus furiosus* Argonaute for the detection of food contaminated bacteria

Yiheng Shi^a^, Pei Gao^a^, Di Wu^b^, Yongning Wu^a,c^,Guoliang Li^a*^

a. School of Food Science and Engineering, Shaanxi University of Science & Technology, Xi’an 710021, P. R. China.

b. Institute for Global Food Security, School of Biological Sciences, Queen's University Belfast, 19 Chlorine Gardens, Belfast, BT9 5DL, United Kingdom

c. NHC Key Laboratory of Food Safety Risk Assessment, Food Safety Research Unit (2019RU014) of Chinese Academy of Medical Science, China National Center for Food Safety Risk Assessment, Beijing 100021, China

***CORRESPONDING AUTHOR:**

E-mail: [61254368@163.com](mailto:61254368@163.com)

| Foodborne pathogens | Oligo name | Sequences(5’-3’) |
| --- | --- | --- |
| *E.coli* O157:H7 | F1 | CGTTAATACGGICAACAAATACTTTCTACCG |
|  | R1 | CTGAACTCCATTAACGCCAGATATGATGAAAC |
|  | F1’ | CGTTAATACGGCAACAAATACTTTCTACCG |
| *S. aureus* | nuc-F2 | GTAGCTTCAAGTCITAAGTAGCTCAGCAAAT |
|  | nuc-R2 | GGTGTATCAACCAATAATAGTCTGAATGTC |
| *S. typhimurium* | F3 | GTGCTTGAATACCIGCCTGTCACAGGTTCAGAGC |
|  | R3 | GGTGCGCGAACTTGTGGTCCTTTTCCAGATTAC |
| *C. sakazakii* | F4 | GTAATGAGTGAAIAGGCGTTACCGATTGATA |
|  | R5 | TCGCAGATTAGCACGTCCTTCATCGCCTCT |
|  | *E. coli* O157:H7-reporter | FAM-cgcaccGAAAAGTCTATCGTAAggtgcg-BHQ1 |
|  | *S. aureus*- reporter | FAM-cgcaccATTTGCTGAGCTACTTggtgcg-BHQ1 |
|  | *S. typhimurium* - reporter | JOE-cgcaccGCTATAACACAGTTTAggtgcg-BHQ1 |
|  | *C. sakazakii*- reporter | JOE-cgcaccATCAATCGGTAACGCCggtgcg-BHQ1 |

**Table S1** Oligonucleotide sequences

**
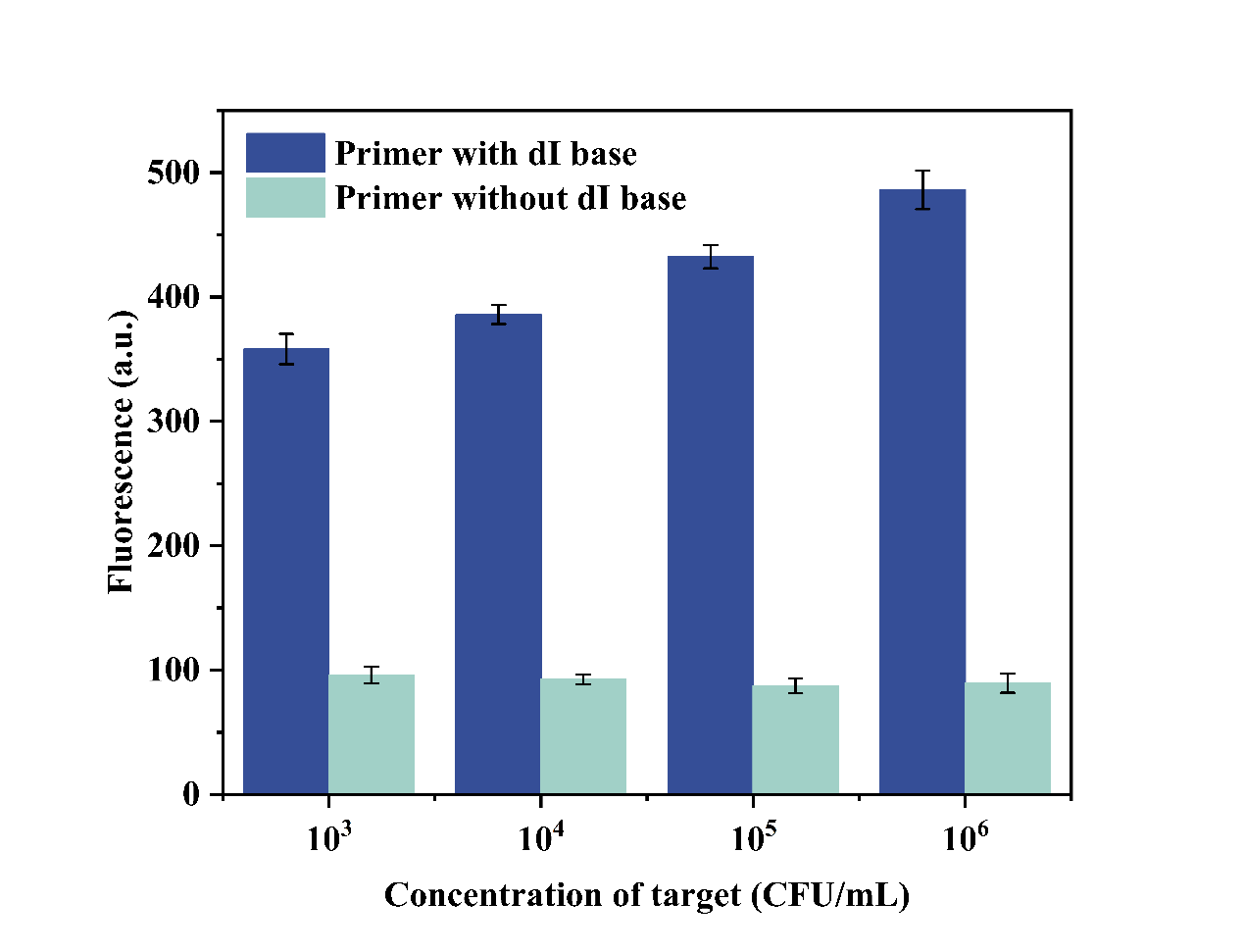
**

Figure S1. The VPN detection system without dI base in RPA amplification primers was compared with the RPA amplification primers containing dI base. （source: Origin）


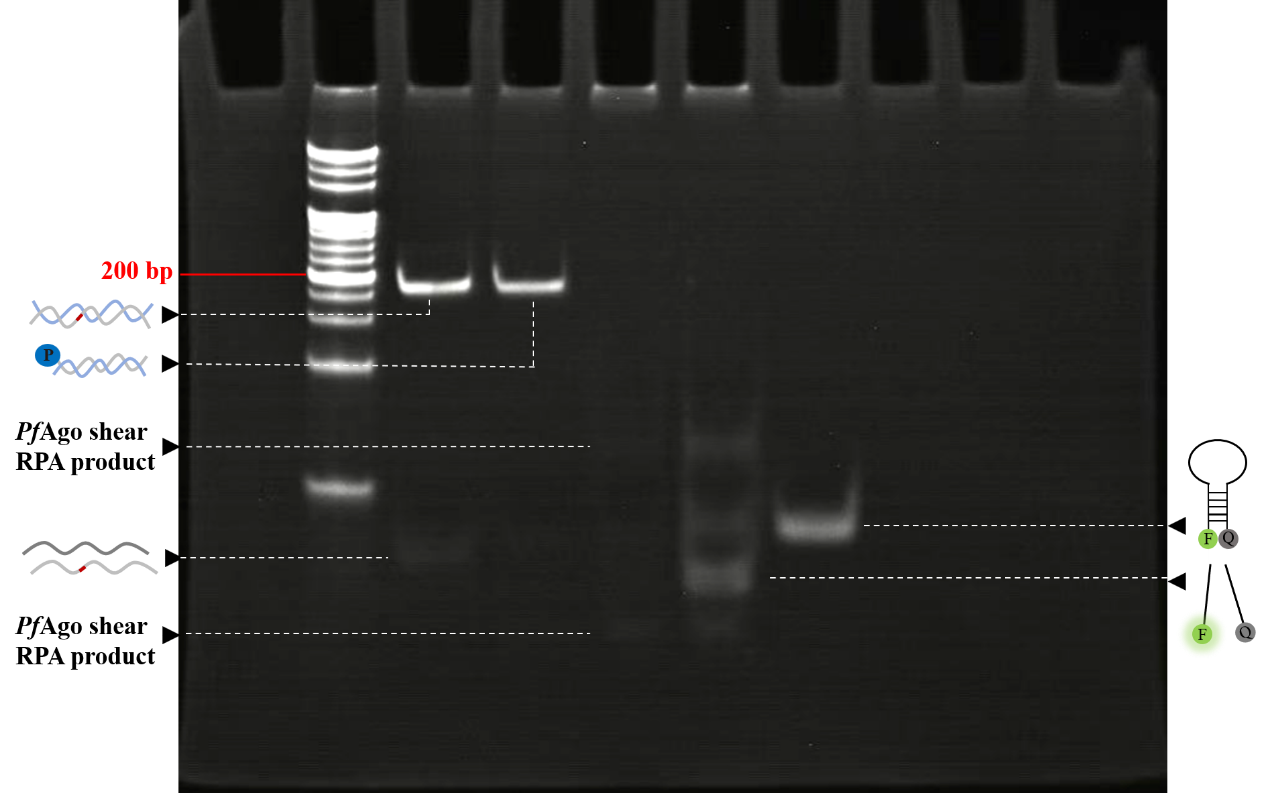


Figure S2. Native polyacrylamide gel electrophoresis (15%). (line 1:amplification product, line 2: Endo V cleave amplification product, line 3: in the absence of MB, *Pf*Ago cleave Endo V cleavage products, line 4: PfAgo cleave Endo V cleavage products with MB, line 5: MB) （source: Microsoft Office PowerPoint, Origin）

**
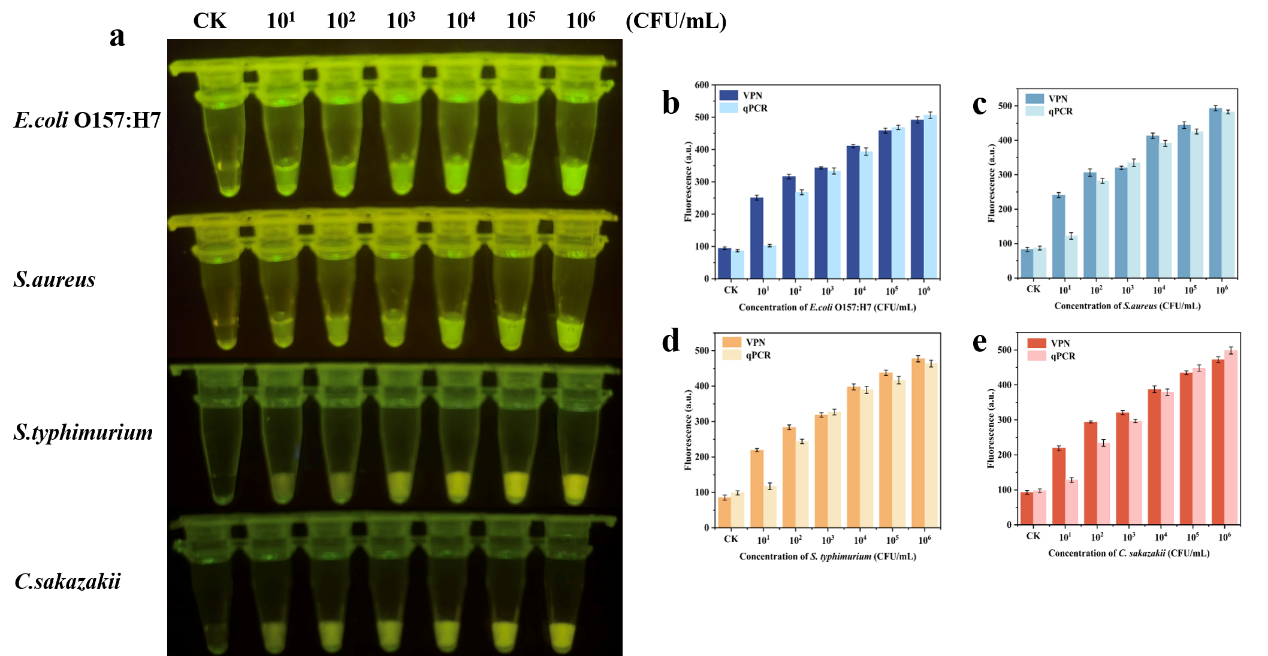
**

Figure S3. Application of VPN detection system. (**a**) Fluorescence images of spiked samples under 365 nm UV lamp. (**b-e**) Comparison of food-contaminated bacteria in real samples (beef, milk) detected by VPN detection system and qPCR. （source: Microsoft Office PowerPoint, Origin）
